# Management of the arrhythmic manifestations of cardiac sarcoidosis

**DOI:** 10.3389/fcvm.2023.1104947

**Published:** 2023-05-25

**Authors:** Callum Cherrett, William Lee, Nicole Bart, Rajesh Subbiah

**Affiliations:** ^1^ Cardiology Department, St Vincent’s Hospital Sydney, Sydney, NSW, Australia; ^2^ School of Medicine, University of New South Wales, Sydney, NSW, Australia; ^3^ Victor Chang Cardiac Research Institute, Sydney, NSW, Australia

**Keywords:** cardiac sarcoidosis, electrophysiology, immunosuppression, atrioventricular block, ventricular tachycardia

## Abstract

Cardiac sarcoidosis (CS) is characterised by a high burden of arrhythmic manifestations and cardiac electrophysiologists play an important role in both the diagnosis and management of this challenging condition. CS is characterised by the formation of noncaseating granulomas within the myocardium, which can subsequently lead to fibrosis. Clinical presentations of CS are varied and depend on the location and extent of granulomas. Patients may present with atrioventricular block, ventricular arrhythmias, sudden cardiac death or heart failure. CS is being increasing diagnosed through use of advanced cardiac imaging, however endomyocardial biopsy is often still required to confirm the diagnosis. Due to the low sensitivity of fluoroscopy-guided right ventricular biopsies, three-dimensional electro-anatomical mapping and electrogram-guided biopsies are being investigated as a means to improve diagnostic yield. Cardiac implantable electronic devices are often required in the management of CS, either for pacing or for primary or secondary prevention of ventricular arrhythmias. Catheter ablation for ventricular arrythmias may also be required, although this is often associated with high recurrence rates due to the challenging nature of the arrhythmogenic substrate. This review will explore the underlying mechanisms of the arrhythmic manifestations of CS, provide an overview of current clinical practice guidelines, and examine the important role that cardiac electrophysiologists play in managing patients with CS.

## Introduction

Sarcoidosis is a systemic inflammatory disease characterised pathologically by the formation of noncaseating granulomas (1). It most often affects the lungs and lymph nodes, but the heart, liver, spleen, skin, eyes and parotid glands can also be involved (1–3). Based on autopsy studies, cardiac involvement is seen in up to 25%–69% of those with sarcoidosis, although this may only be clinically manifest in 5% (4–6). Epidemiological data from Finland estimate an annual detection rate of 0.31 per 10^5^ adults and prevalence of 2.2 per 10^5^ adults. Between 1988 and 2012 there was a >20-fold increase in histologically confirmed cases of cardiac sarcoidosis (CS). The higher rate of detection is thought to be due to heightened awareness and improved diagnostic imaging (7). Cardiac sarcoidosis may result in conduction abnormalities, tachyarrhythmias, sudden cardiac death and heart failure, with the manifestations dependent on the location, extent and activity of the disease (5, 8). Cardiac involvement is responsible for a significant burden of morbidity and mortality in sarcoidosis (9). To date, management recommendations are primarily based upon consensus and expert opinion, given the lack of randomised controlled trial data (3). Cardiac sarcoidosis is challenging to diagnose as it can mimic a number of phenotypically similar cardiac syndromes (3, 10). A multidisciplinary team approach, including heart failure cardiologists, electrophysiologists, and imaging specialists, is recommended in the diagnosis and management of patients with suspected CS (3, 8, 10, 11). This review article will focus on the arrhythmic manifestations of CS and the role of the cardiac electrophysiologist in this multidisciplinary team.

## Pathogenesis of cardiac sarcoidosis

The formation of discrete noncaseating granulomas is the pathological hallmark of sarcoidosis (12). Granuloma formation is thought to result from exposure to an unknown antigen leading to an exaggerated immune response in patients with an underlying genetic susceptibility (3). The majority of patients with sarcoidosis (90%–95%) have pulmonary and lymph node involvement, but isolated CS can occur. In CS, granulomas typically develop in the myocardium, often with endocardial or epicardial extension (9). The extent and distribution of these granulomas dictate the clinical manifestations. Often there is a predilection for involvement of the basal septum resulting in atrioventricular block and distal conduction abnormalities (3). Active myocardial inflammation can result in ventricular arrhythmias, ventricular dysfunction and sudden cardiac death. Furthermore, in the post-inflammatory phase, patchy fibrosis in the ventricular myocardium can develop, further predisposing to ventricular tachyarrhythmias (9).

## Electrophysiological manifestations of cardiac sarcoidosis

Cardiac sarcoidosis is characterised by a high burden of arrhythmic manifestations. High-grade atrioventricular (AV) block was found to be the most common first manifestation of CS (presenting feature in 42% of cases) in an analysis of 351 patients with CS in Finland between 1998 and 2015. Heart failure (17%) was the next most common first presentation of CS, following by fatal or aborted SCD (14%) and sustained ventricular tachycardia (14%) (13). Analysis of 161 patients from the Cardiac Sarcoidosis Consortium who underwent at least one 24-h Holter monitor revealed the presence of high-degree AV block in 5% of patients, non-sustained ventricular tachycardia (NSVT) in 34% and sustained ventricular tachycardia (VT) in 4%. Premature ventricular contraction burden (PVC) of ≥5% was seen in 22% of patients and a burden ≥20% in 6% (14). Data from the ILLUMINATE-CS registry, which included 512 patients diagnosed with CS between 2001 and 2017 revealed the estimated 5- and 10-year fatal ventricular arrhythmia event rates to be 20.7% and 31.9%, respectively (15).

It is important to consider and screen for CS in patients presenting with unexplained AV block or ventricular arrhythmias. In a study of 32 patients aged 18–60 presenting with unexplained 2nd or 3rd degree AV block and no previous history of sarcoidosis, 11/32 (34%) had CS following investigation with advanced cardiac imaging (FDG-PET ± CMR) (16). Similarly, in patients presenting with ventricular arrhythmias, excluding patients with active ischaemic heart disease or known sarcoidosis, 5%–28% had CS diagnosed following investigation with FDG-PET ± CMR (17, 18).

Supraventricular arrhythmias (SVA) are also seen in up to 32% of patients with CS (19). SVA may result from increased end-diastolic pressure associated with the left ventricular dysfunction seen in patients with advanced CS, or due to granulomatous involvement of the atria (12). In a study of 118 patients with CS and no prior history of atrial fibrillation, 34 patients (29%) had paroxysmal AF during median follow up of 3 years, with 7 patients developing persistent AF and 4 patients developing permanent AF (20). ^18^F-FDG uptake in the atrium at the time of diagnosis of CS, was found to be an independent predictor of future atrial fibrillation (HR of 6.01, 95% CI: 2.64–13.66) (20).

It is often difficult to differentiate cardiac sarcoidosis from other conditions such as arrhythmogenic right ventricular cardiomyopathy (ARVC) or myocarditis (21). In a prospective study of patients with left bundle branch block (LBBB)-pattern VT and suspected ARVC, 15% were subsequently diagnosed with CS following endomyocardial biopsy (22). In another cohort of patients with LBBB-pattern VT with either ARVC or CS who were referred for radiofrequency catheter ablation, 5/8 (63%) of patients with CS fulfilled the diagnostic ARVC criteria (23). Recently a novel electrocardiogram (ECG)-based algorithm to differentiate CS from ARVC in patients presenting with right ventricular (RV) VT was developed, by assessing differences in terminal activation in leads V1–V3 (24). It is hypothesised that the RBBB pattern seen in ARVC results from fibro-fatty infiltration predominantly in the RV causing diffuse conduction delay and reduced voltages, compared to the focal and patchy granulomas in CS causing local block and preserved voltages reflected by an R’ wave with a higher voltage. A PR interval ≥200 ms and/or the presence of an R' wave with a maximum surface area in V1–V3 ≥1.65 mm identifies patients with CS rather than ARVC with a sensitivity of 83% and specificity of 88% in their validation cohort (24).

## Screening of patients with extra-cardiac sarcoidosis

Autopsy studies of patients with sarcoidosis have found evidence of cardiac involvement in up to 69.1% of cases (6). As symptomatic cardiac involvement may only be manifest in 5% of patients, the Heart Rhythm Society (HRS) 2014 guidelines recommend screening for patients with extra-cardiac sarcoidosis (5). It is recommended that symptoms of syncope/presyncope/palpitations should be sought for on history and all patients should undergo a 12-lead ECG. Among 227 patients with extra-cardiac sarcoidosis, 56 (25%) had ECG abnormalities (25). Conduction abnormalities, including prolonged PR interval, right bundle branch block, left anterior hemiblock and left bundle branch block were seen in 23 patients (10%), 23 patients (10%) had a fragmented QRS complex and ST-T abnormalities were seen in 19 patients (18%). Eleven patients (4.8%) experienced a cardiac event (either AV-block, VT or heart failure) during a follow up period of 6.2 years. All patients were found to have an ECG abnormality prior to their cardiac event. The HRS 2014 guidelines also state that echocardiography can be useful in screening patients with extra-cardiac sarcoidosis and that advanced cardiac imaging (CMR or FDG-PET) can be useful in patients with one or more abnormalities detected on initial screening with history/ECG/echocardiography (1).

Signal-averaged electrocardiography (SAECG) may also be useful for early detection of CS in patients without intraventricular conduction delay (26–30). SAECG can detect late potentials, which are low-amplitude, high-frequency signals at the terminal portion of the QRS, which reflect heterogeneous and fragmented activation of diseased ventricular myocardium (26, 29). In one study of 74 patients with pulmonary sarcoidosis, 29 (39.2%) had detectable late potentials. At 9.8 years of follow up, 8/29 (28%) patients with late potentials had a cardiovascular event (AV-block, VT or heart failure) compared to only 1/45 without detectable late potentials (28). Wavelet-transformed ECG (WTECG) is also under investigation as a potential tool to detect arrhythmogenic substrate in patients with sarcoidosis. WTECG can be used to detect high-frequency components, which reflect slow conduction through affected myocardium. Although the use of WTECG has only been evaluated in one retrospective study of patients with CS, it does have the potential benefit of being used in patients with intraventricular conduction delay, unlike SAECG (29).

Screening for CS in patients with extra-cardiac sarcoidosis may identify patients who are at risk of VA and sudden cardiac death (SCD). Bakker et al., reported their experience of screening patients with extra-cardiac sarcoidosis and subsequent rates of VA and SCD. In this study, 114/547 (21%) patients were diagnosed with CS following assessment with advanced cardiac imaging. Nine patients were lost to follow up. Implantable cardioverter-defibrillators were subsequently implanted in 17/105 patients (16.2%) who were considered high risk for SCD. Eighty patients (76.2%) who were considered low risk for SCD had an implantable loop recorder (ILR), which was a local recommendation for patients without an ICD indication. High and low risk patients were identified based on the HRS guidelines, LV ejection fraction and the presence and extent of LGE on CMR. At a follow-up period of 33 months, sustained ventricular arrythmias, appropriate ICD therapy and cardiac death occurred in 4.8%, with an annualized event rate of 1.7%. In patients who were considered low risk and underwent ILR insertion, 15% of patients had clinically important arrhythmias detected; 11/80 had NSVT, 2/80 had AVB, and there was 1 cardiac death (non-arrhythmic; due to right sided cardiac failure) (31).

## Establishing a diagnosis of cardiac sarcoidosis

**Figure 1:**
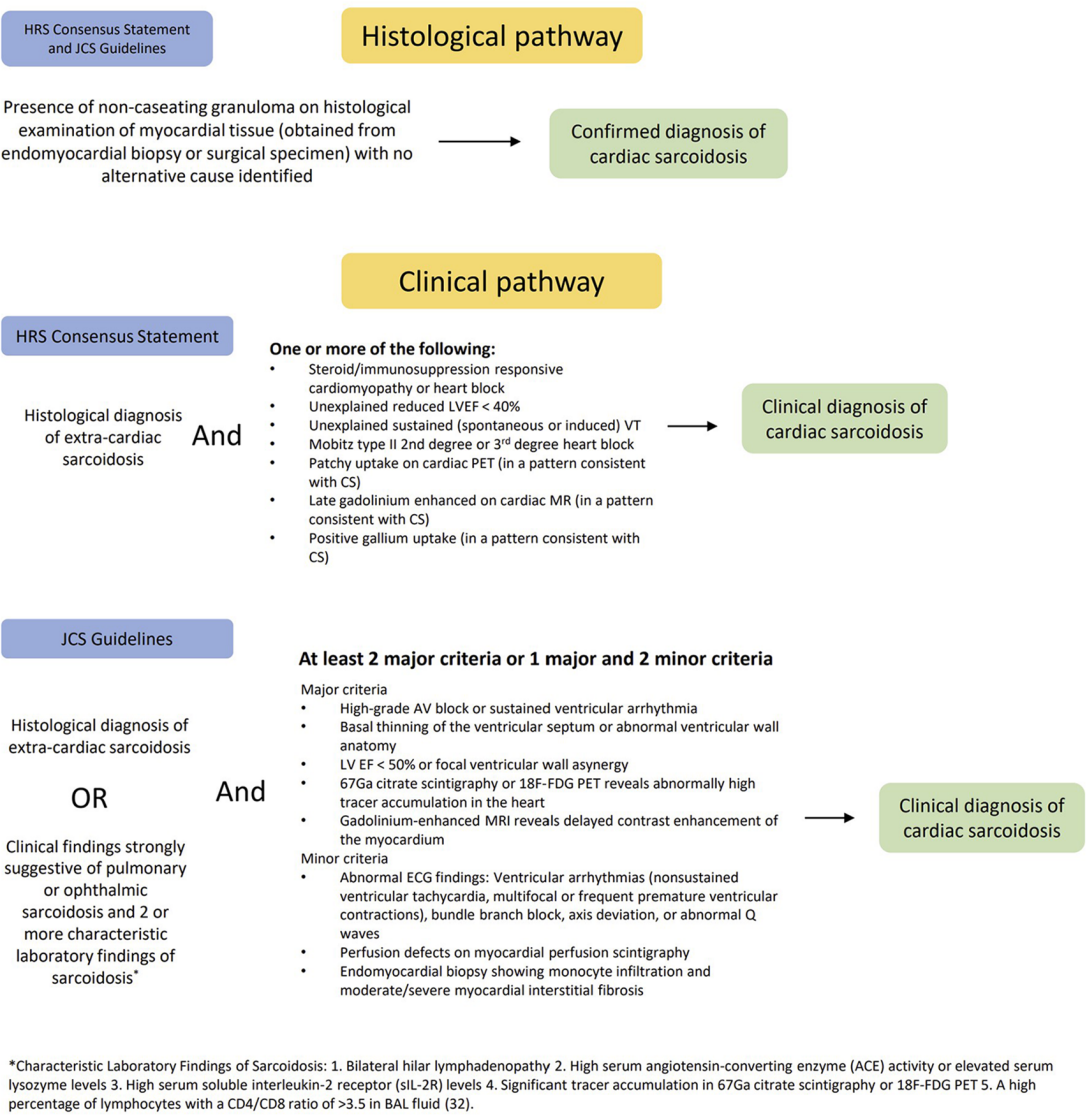
Cardiac sarcoidosis diagnostic criteria outlined in the HRS 2014 consensus statement and JCS criteria.

There are two commonly used guidelines, the Heart Rhythm Society (HRS) 2014 consensus statement and the Japanese Circulation Society (JCS) criteria, to establish a diagnosis of CS as illustrated in Figure 1 (1, 32). Both guidelines stipulate that a histological diagnosis is established by the presence of non-caseating granulomas in myocardial tissue (with no alternative cause identified). However, due to the patchy nature of CS, the sensitivity of endomyocardial biopsies is low and a diagnosis of CS is not excluded by a negative biopsy result (3). The HRS and JCS guidelines also set out a clinical diagnosis pathway, which is met when there is a histological diagnosis of extra-cardiac sarcoidosis in conjunction with non-invasive evidence of cardiac involvement. In addition to a histological diagnosis of extra-cardiac sarcoidosis, the HRS 2014 guidelines also require one of more of the following to establish a “probable” diagnosis of CS: steroid ± immunosuppressant responsive cardiomyopathy or heart block, unexplained reduced LVEF (<40%), unexplained sustained (spontaneous or induced) ventricular tachycardia (VT), Mobitz type II 2nd degree or 3rd degree AV block, or findings on advanced cardiac imaging in a pattern consistent with CS (patchy uptake on cardiac Positron Emission Tomography (PET), Late Gadolinium Enhancement (LGE) on Cardiac Magnetic Resonance Imaging (CMR), positive gallium uptake) (1).

## Endomyocardial biopsy moving to the cardiac electrophysiology laboratory

**Figure 2:**
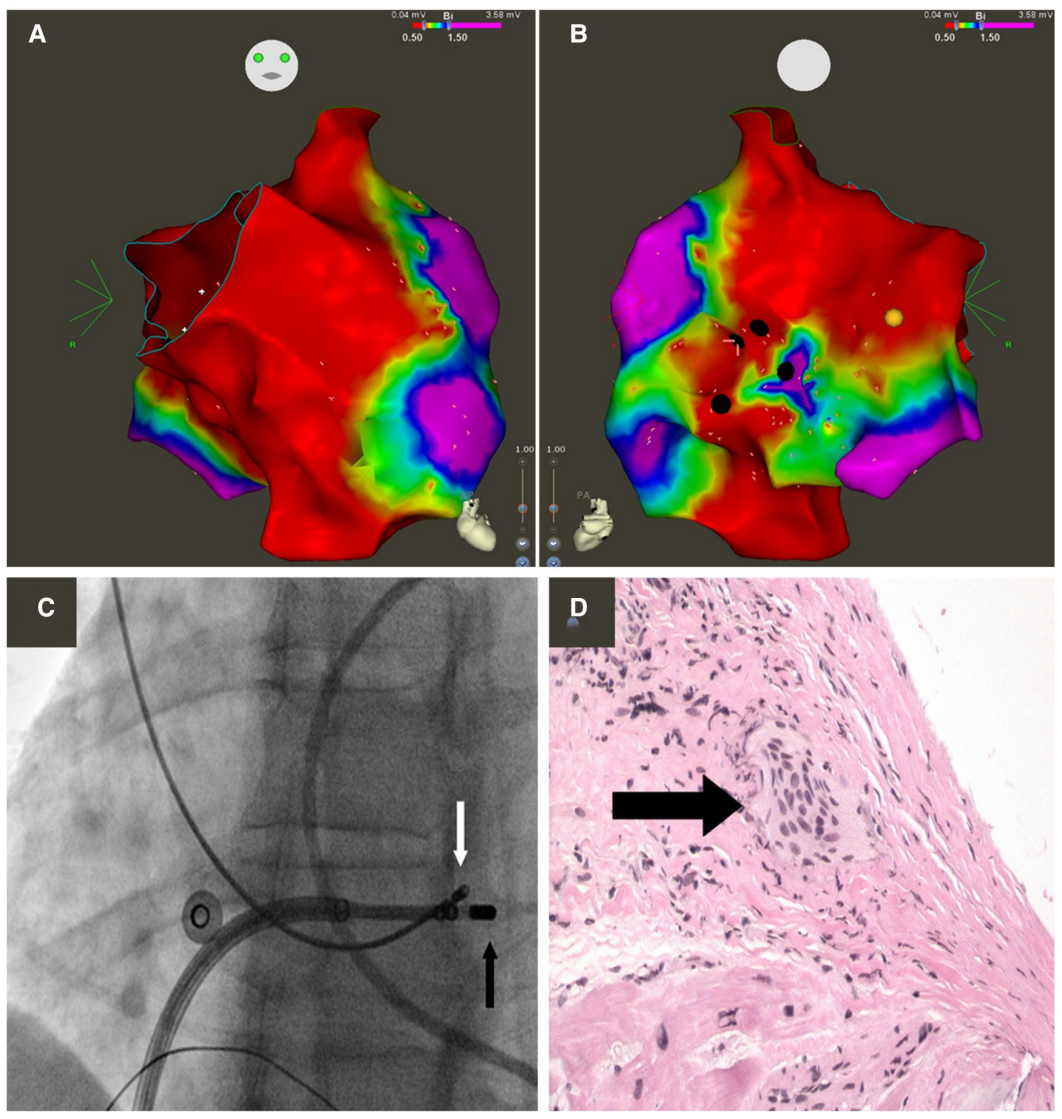
Electroanatomic bipolar voltage map of the right ventricle displaying anterior (**A**) and posterior (**B**) views. Green, yellow, and red indicate low-voltage regions; purple denotes regions of normal voltage, defined as ≥1.5 mV. Black circles illustrate areas targeted for biopsy. Yellow circle illustrates location of right bundle. (**C**) Fluoroscopy images obtained in the left anterior oblique 25° projection showing bioptome (white arrow) targeting the low-voltage regional in the right ventricular septum, adjacent to the mapping catheter (black arrow). (**D**) Microscopic view of an endo-myocardial biopsy specimen obtained from the right ventricular septum showing noncaseating granuloma (arrow). Hematoxylin-eosin; magnification, 200x. Reprinted from Canadian Journal of Cardiology, 29(8):1015.e1–1015.e3, Nery PB, Keren A, Healey J, et al, Isolated cardiac sarcoidosis: establishing the diagnosis with electroanatomic mapping-guided endomyocardial biopsy, 2013, with permission from Elsevier (35).

Endomyocardial biopsy, using three-dimensional (3D) electro-anatomical mapping, may aid in establishing a histological diagnosis of CS. The yield of fluoroscopy-guided right ventricular endomyocardial biopsy in patients with suspected CS is low, which is believed to be due to the heterogenous and patchy distribution of sarcoid granulomas and the tendency for CS to involve the left ventricular (LV) mid-myocardium and sub-epicardium (33, 34). Nery et al. first described the use of electroanatomical mapping-guided endomyocardial biopsy to diagnose CS (Figure 2) (35). Since this description, a number of groups have reported the use of 3D electroanatomical mapping-guided biopsies to detect CS (34, 36, 37). Ezzeddine et al. published the largest cohort of patients using 3D electroanatomical mapping (CARTO, Biosense Webster) to perform RV ± LV electrogram (EGM)-guided biopsies (Figure 2). A total of 79 patients underwent either isolated endomyocardial biopsy or combined endomyocardial biopsy with VT/PVC ablation. Sites targeted for biopsy had abnormal bipolar signals (fractionated EGMs, late potentials and/or low voltage EGMs). Fluoroscopy and intracardiac echocardiography (ICE) were used to direct the bioptome to the sites with abnormal EGMs. If no abnormal endocardial EGM signals were detected, substrate abnormalities on CMR or PET scan were used to guide specimen collection. Following histopathological analysis, 16/79 (20%) had confirmed CS. Ten patients (13%) had an alternative diagnosis identified. Abnormal EGM signals had a sensitivity of 89% and specificity of 33% in predicting CS (34).

## Pacing in cardiac sarcoidosis

Atrioventricular block is a common manifestation of CS and permanent pacing is indicated, even if the AV block reverses transiently (1). There have been case reports of AV block being treated successfully with steroid therapy alone without permanent pacing (38). It has been suggested that patients with FDG uptake in the vicinity of the AV node may predict recovery with corticosteroids, and patients with LGE without active inflammation may predict a poor response to immunosuppression (39). However, as AV recovery is unpredictable and can be transient, permanent pacing is still indicated in these cases (2). Given the potential risk of future ventricular arrhythmias, for those who are ICD candidates, there is a class IIa indication for ICD for all patients requiring permanent pacing (1, 21, 39).

There is limited evidence in the literature supporting the use of cardiac resynchronization therapy (CRT) in CS. At the time of writing the 2014 HRS guidelines, there was no specific data relating to CRT in CS patients. The writing group suggested that findings from the major trials and relevant recommendations should apply to CS patients (1). The largest study is a retrospective analysis of 55 patients with definite (18/55), probable (21/55) or presumed (16/55) CS who underwent CRT implantation at the Mayo Clinic (Arizona, Florida and Minnesota campuses) between 2000 and 2021. Most (67.3%) patients received a device upgrade rather than a *de novo* implantation and so the intrinsic QRS morphology could only be determined in a minority of patients (10.9% had a LBBB, 16.4% had a RBBB, 7.3% had nonspecific ventricular conduction delay). A positive response to CRT (defined as >5% improvement in LVEF) was seen in 23/55 (41.8%) patients at 6 months. In the overall population, there was no significant improvement in left ventricular function (average LV EF 34.8% at baseline, 37.7% at 6 months) or left ventricular end-diastolic dimensions (58.5 mm at baseline, 57.5 mm at 6 months). Poor response to CRT was thought to be due to myocardial scarring and fibrosis in advanced CS (40).

## Sudden cardiac death risk stratification and implantable cardioverter defibrillators

Class I indications for ICD implantation in patients with CS in the 2017 AHA/ACC/HRS guidelines include patients with sustained ventricular tachycardia, those who survive sudden cardiac arrest and patients with an LVEF of ≤35% (21). Implantable cardioverter defibrillator insertion is reasonable (class IIa indication) in patients with CS and LVEF ≥ 35% who present with syncope, who have evidence of myocardial scar by cardiac MRI or PET scan, have a positive EPS or who have an indication for permanent pacing. A device that can also provide bradycardia pacing is preferred over a subcutaneous defibrillator given the frequency of conduction abnormalities (21). It is important to consider when programming ICD detection and therapies, that heart block may resolve with immunosuppressive therapy, and may allow for the rapid conduction of atrial arrhythmias, resulting in inappropriate therapies (1).

Advanced cardiac imaging, particularly CMR, has an important role in prognostication and guiding the use of implantable cardioverter defibrillators. Coleman et al. performed a meta-analysis of 706 patients with known or suspected CS undergoing cardiac MRI. Patients with LGE detected on CMR had significantly higher odds of the combined end-point including arrhythmogenic events (ventricular arrythmias, sudden cardiac death, appropriate implantable cardioverter defibrillator therapy) and all-cause mortality (OR: 10.74, *p* < 0.00001). In studies of patients with LVEF ≥ 50%, the association of LGE with the composite end-point was greater (OR: 19.43, *p* < 0.00001). The annualized event rate of the composite outcome for patients with LGE was 11.9% compared to 1.1% in patients without LGE (41).

Patterns of myocardial involvement frequently seen in advanced CS, as assessed by gross pathological specimens in patients who underwent autopsy or transplantation, include LV subepicardial, LV multifocal, septal and RV free wall (these patterns are termed pathology-frequent). Pathology-rare patterns (which include lack of gross LV myocardial involvement, isolated LV midmyocardial involvement, isolated LV subendocardial involvement, isolated transmural involvement or isolated involvement of only one LV level) were rarely seen in advanced CS (42). Pathology-frequent LGE was associated with a higher risk of ventricular arrhythmias and heart failure, independent of LVEF and extent of LV LGE. A combined arrhythmic endpoint, including sudden cardiac death, resuscitated cardiac arrest with documented ventricular arrhythmias (VA), sustained VT and appropriate implantable cardioverter defibrillator (ICD) therapy, was met in 28.2% of patients with pathology-frequent LGE compared to 0.0% for patients with pathology-rare LGE (43).

A large retrospective cohort study has shown that the class I and class IIa indications for ICD implantation in the 2017 AHA/ACC/HRS guidelines identified all patients with a composite endpoint of significant ventricular arrhythmia or sudden cardiac death at a median follow-up of 3 years. This study included 290 patients with known or suspected CS who underwent cardiac MRI. At 1.9 years, the Kaplan-Meier estimate of the cumulative incidence of the composite endpoint (significant ventricular arrhythmia or sudden cardiac death) in patients with a class I indication was 52.6%, 18% for those meeting any class IIa indication and 0% for those without an ICD indication. In patients without a class I indication for ICD, the optimal cutoff using ROC analysis of LGE extent to predict the composite endpoint was 5.7%. A LGE burden of >5.7% had a significantly improved specificity (94.6%) without affecting sensitivity. Using a cut-off of 5.7% for LGE burden in this cohort would have reduced the number of patients eligible for ICD implantation by 73% (44).

A similar study by Nordenswan et al., examined 398 patients over a longer median follow up period and showed that in patients with Class I and IIa indications by the 2017 AHA/ACC/HRS guidelines, the 5-year incidence of SCD or sustained VT was 24.7%. Patients without Class I or IIa indications by the 2014 HRS guidelines (which did not include evidence of myocardial scar on CMR or PET) had a 5-year risk of SCD of 4.8% and a 5-year risk of SCD or sustained VT of 12.1%. By the 2014 HRS guidelines, in patients without ICD indications at presentation, the 5-year incidence of SCD, sustained VT and emerging Class I or IIa indications was 53%, however by the ACC/AHA/HRS 2017 guidelines, all 245 patients had a Class I or IIa indication for ICD implantation. This clearly illustrates the importance of utilising advanced cardiac imaging to identify areas of scar in CS, which may predispose to ventricular arrhythmias and SCD, in the absence of other indications for ICD (45).

An electrophysiology study (EPS) with programmed ventricular stimulation can be used to risk stratify patients who do not otherwise meet criteria for ICD implantation. The use of EPS in patients with CS and LVEF >35% without arrhythmic symptoms or documented arrhythmias is supported by the 2017 AHA/ACC/HRS guidelines (class IIa recommendation) and the JCS 2016 guidelines (21, 32). A meta-analysis, which included 8 studies and 298 patients, evaluating the utility of EPS in predicting future ventricular arrhythmias or SCD found the sensitivity to be 0.70 (0.51–0.85) and specificity 0.93 (0.85–0.97) (46). However, many of those 8 studies included patients with LVEF < 35% whom would already satisfy ICD implantation criteria. There are two observational studies that provide specific outcomes for CS patients with LVEF > 35%, where the use of EPS as a risk stratification tool is currently recommended (46). Zipse et al. performed an EPS on 120 patients with extra-cardiac sarcoidosis and LVEF ≥ 50%. Seven of 120 (6%) had a positive EPS and received an ICD. Three patients received appropriate ICD therapies for VT during follow up, 1 of these patients later died in the setting of electrical storm (47). One patient with a negative EPS had sustained VT on routine telemetry 1 year after EPS and received an ICD. A second patient with a negative EPS died suddenly weeks after a flare of pulmonary sarcoid (CS was confirmed on postmortem histology) (47). Okada et al. reported 2/12 of patients with “probable CS” or “definite CS” with LVEF > 35% and no prior ventricular arrhythmias had a positive EPS. Both patients with a positive EPS developed VAs whereas 1/10 patients with a negative EPS had VA in the follow up period. When combining these two studies in the meta-analysis subgroup of LVEF > 35%, Adhaduk et al. reports a specificity of 0.97 (0.92–0.99), sensitivity of 0.63 (0.29–0.99), positive predictive value of 0.56 (0.29–0.79) and negative predictive value of 0.98 (0.94–0.99) for EPS as a risk stratification tool in CS (46).

## Effects of immunosuppression on arrhythmias associated with cardiac sarcoidosis

**Figure 3:**
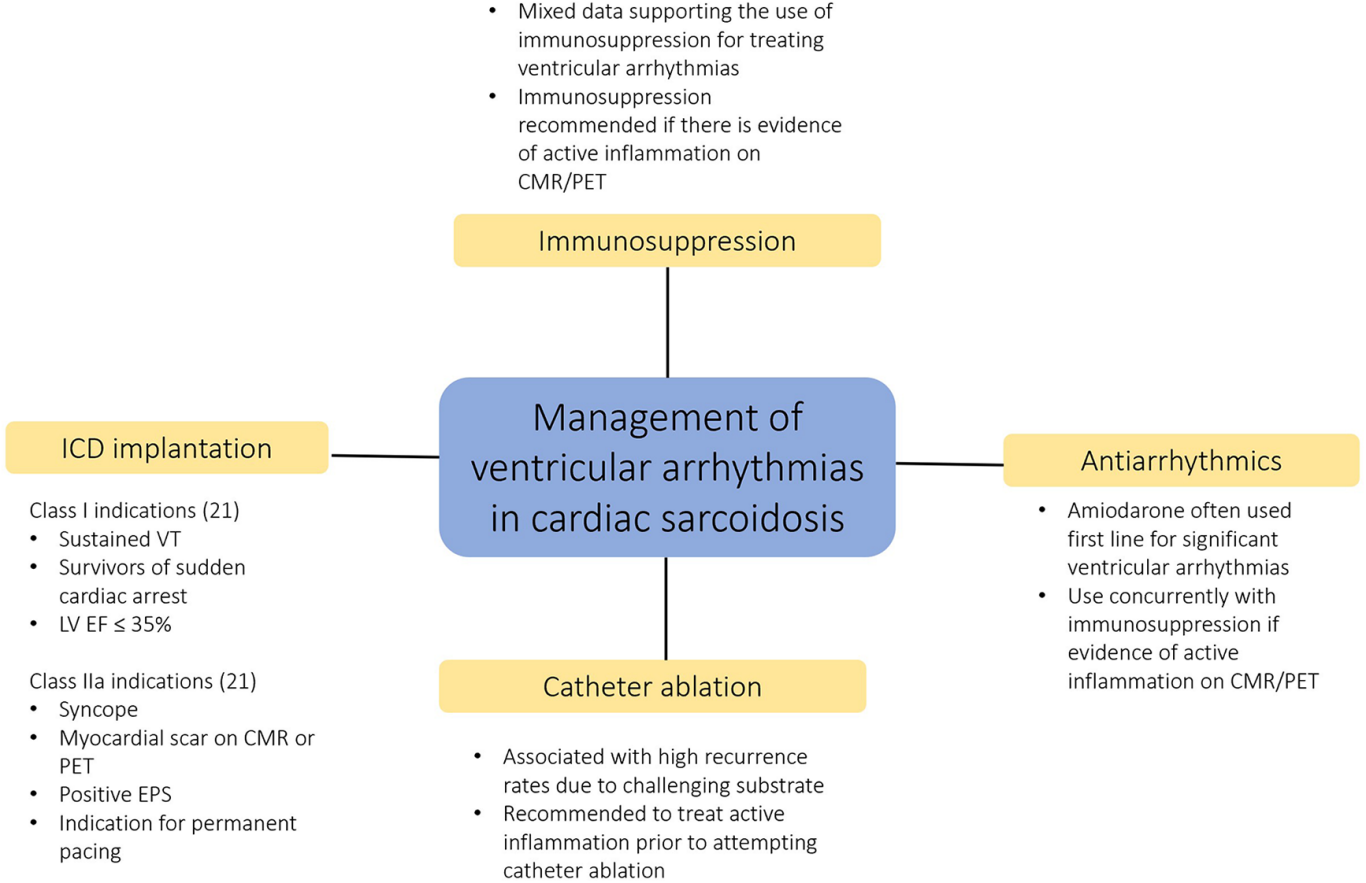
Multi-faceted approach to the management of ventricular arrhythmias in cardiac sarcoidosis.

Management of ventricular arrhythmias in cardiac sarcoidosis often requires a combination of immunosuppressive therapy, antiarrhythmic therapy, device implantation and in some cases catheter ablation (Figure 3) (3).

Based on retrospective studies and consensus opinion, immunosuppression with glucocorticoids is recommended as first-line therapy to reduce active inflammation and prevent irreversible myocardial fibrosis and remodeling in CS (3, 12). Following an initial period of glucocorticoid use, as immunosuppressive therapy may be required for years, steroid-sparing agents such as methotrexate, azathioprine, mycophenolate or biologic agents are often introduced (12). To date, there are no randomised controlled trials investigating the effects of immunosuppression for CS. A meta-analysis of 13 observational studies reported the outcomes of immunosuppression use in CS patients with high grade AV block. Of 199 patients, 178 (89%) patients received corticosteroids or other immunosuppressants. Of these 178 patients who received immunosuppression, 76 (42%) had recovery of AV node conduction. No patients without immunosuppression had AV conduction recovery (2).

Eight studies including in the meta-analysis also assessed the effects of immunosuppression on ventricular arrhythmias, but concluded the data was too limited to draw any conclusions (2). In total, 69 patients in this meta-analysis were treated with corticosteroids for sustained ventricular arrythmias, but most patients also had concurrent antiarrhythmics or catheter ablation. Arrhythmia recurrences rates ranged between 14% and 71%. Three studies have also examined the effects of immunosuppression on premature ventricular contraction (PVC) burden, two studies showing no significant difference before and after steroids, with one study actually showing an increase in PVC burden following corticosteroids. Medor et al. reported an increase in PVC burden on ICD interrogation in 18 of 20 patients (90%) with active CS on FDG-PET who were treated with corticosteroids. On average, there was a threefold increase in daily PVC count as well as a significant increase in episodes of non-sustained VT (48).

VA in CS are predominately thought to arise from macroreentrant circuits around areas of granulomatous scar, rather than triggered activity or enhanced automaticity (seen with active inflammation) which may explain the mixed results seen with immunosuppression for VA (1, 11). Few studies have examined the disease activity with gallium or FDG-PET at the time of initiating immunosuppression for VA (2). Active inflammation may promote VA by slowing conduction in diseased tissue or by triggering ventricular ectopic beats. Therefore, initial treatment of significant VA with antiarrhythmics (usually amiodarone) and concurrent immunosuppression (if there is evidence of active inflammation on cardiac imaging, or empirically if the setting does not permit imaging) is recommended (1).

## Ablation of ventricular arrhythmias associated with cardiac sarcoidosis

Catheter ablation for VA in patients with CS is often associated with higher recurrence rates, similar to other forms of non-ischaemic cardiomyopathy, due to the challenging underlying arrhythmogenic substrate (49). VT in CS is often characterised by multiple different morphologies owing to the diffuse and heterogenous nature of the disease (50, 51). Eliminating all inducible VTs is difficult, endocardial mapping of both ventricles is frequently required and epicardial mapping and ablation is needed in a significant proportion of cases (50, 51). Active inflammation may also promote VT by triggered activity or abnormal automaticity (52). Active inflammation at baseline detected by FGD-PET is associated with increased risk of recurrent VT following catheter ablation, and it is recommended to treat the active disease phase with immunosuppression prior to attempting catheter ablation (52). If there is active inflammation, the arrhythmia substrate is changeable, furthermore oedema may also limit the penetration of thermal energy, further increasing risk of recurrence (11).

A meta-analysis of 401 patients from 15 studies, 95% of who were on antiarrhythmic drugs and 79% on immunosuppressants, showed a 57% acute procedural success rate, with 25% of patients requiring a repeat ablation procedure. All patients underwent endocardial ablation with 23% also undergoing epicardial VT ablation. VT recurrence after first ablation was seen in 214/401 (55%) and after multiple ablations was 81/220 (37%). The combined end-point of death, heart transplant or left-ventricular assist device was met in 21% of patients undergoing VT ablation (53).

Electroanatomical mapping in a cohort of 21 patients undergoing VT ablation for cardiac sarcoid revealed a significant proportion of patients had extensive and confluent regions of endocardial and epicardial right ventricular scarring. In contrast, VT substrate in the LV tended to be patchy, affecting primarily to septum, anterior wall and perivalvular regions. A median of 3 VTs were inducible, with at least 1 VT abolished in 19/21 patients (91%). All VTs were abolished in 9 of 21 patients (43%), with 9 of 21 patients requiring a second procedure. Failure to abolish all VTs was determined to be due to extensive RV scarring, intramural circuits, and circuits in close proximity to epicardial structures (e.g., left anterior descending artery) making ablation at those sites unsafe (50).

## Future directions

Recently, artificial intelligence and deep learning has been applied to ECGs to screen for conditions including heart failure with reduced ejection fraction, hypertrophic cardiomyopathy and cardiac amyloidosis (CA) (54–56). Schrutka et al. used machine learning to analyse a complex data set of electroanatomical maps obtained from electrocardiographic imaging (ECGI) of patients with CA. Characteristics patterns on ECGI were then correlated with visually perceptible surface ECG features, which lead to significant improvements in the detection rate of CA with an area under the curve of 0.97 after training (55). To date, machine learning has not yet been applied to ECG screening of patients with suspected CS, but these preliminary experiences of using machine learning to evaluate ECGs appears promising.

There are several approaches that are being investigated to improve VT ablation in non-ischaemic cardiomyopathies. As is seen in other forms of non-ischaemic cardiomyopathy, many patients with CS have intra-mural substrate that is difficult to ablate from an endocardial approach (50). Techniques that have been proposed to enable deeper lesion formation include the use of half-normal saline irrigation, impedance modulation, or the use of a needle-tipped electrode (57). Pulsed field ablation, which has the advantage of tissue specificity, is another exciting technology that may permit effective ablation lesions without risking injuries to adjacent structures such as the coronary arteries (8).

## Conclusion

Cardiac sarcoidosis remains a complex and challenging condition to manage. It is characterised by a high burden of arrhythmic manifestations including AV block, ventricular arrythmias and sudden cardiac death. Identifying patients with CS remains difficult and it is vital moving forward that we continue to improve our diagnostic strategies. Given the patchy nature of the sarcoid granulomas, utilising three-dimensional electro-anatomical mapping and EGM-guided biopsies may increase the diagnostic yield of endomyocardial biopsy. Whilst immunosuppression remains a key component of CS management to reduce myocardial inflammation, some patients will require electrophysiological interventions such as device implantation. There is strong evidence that advanced cardiac imaging, particularly CMR, improves sudden cardiac death risk stratification and is valuable in guiding the use of CIEDs. Whilst current clinical practice guidelines are based on consensus and expert opinion, we await the results of the first randomised controlled trials of immunosuppression for CS, which have begun enrollment. Over the coming years, we are likely to see further development of technologies that will enhance our ability to treat recurrent ventricular arrhythmias with catheter ablation.
